# Biomimetic nanovaccines with self-adjuvant effects induced broad-spectrum neutralizing antibodies against SARS-CoV-2 infection in rodents

**DOI:** 10.1128/jvi.00315-25

**Published:** 2025-10-10

**Authors:** Weiqi Wang, Pengye Du, Yongkun Zhao, Yuan Liang, Cheng Zhang, Hongjie Zhang, Xianzhu Xia, Bo Liu, Pengpeng Lei, Feihu Yan

**Affiliations:** 1 Changchun Veterinary Research Institute, Chinese Academy of Agricultural Sciences 595703 , Changchun, Jilin, China; 2 University of Science and Technology of China 12652, Hefei, Anhui, China; 3 State Key Laboratory of Rare Earth Resource Utilization, Changchun Institute of Applied Chemistry, Chinese Academy of Sciences 58277, Changchun, Jilin, China; 4 Department of Microorganism Engineering, Beijing Institute of Biotechnology, Beijing, China; University of North Carolina at Chapel Hill, Chapel Hill, North Carolina, USA

**Keywords:** SARS-CoV-2, biomimetic nanovaccines, broad-spectrum, cross-neutralizing antibody, cellular immunity

## Abstract

The emergence of severe acute respiratory syndrome coronavirus-2 (SARS-CoV-2) variants seriously threatens the efficacy of current coronavirus disease 2019 (COVID-19) vaccines. Therefore, there is an urgent need to develop next-generation vaccine platforms capable of counteracting current and prospective viral variants. In this study, we employed synthetic biology technology to develop self-adjuvant-effect biomimetic nanovaccines with broad-spectrum capabilities for the prevention of SARS-CoV-2 infection. The biomimetic nanovaccines prepared by loading the SARS-CoV-2 RBD protein into dendritic mesoporous organosilicon nanoparticles (DMOSN) significantly promote the recruitment of dendritic cells (DCs) to secondary lymphoid organs, thereby initiating antibody-dependent humoral immune responses mediated by follicle helper T (Tfh) cells, germinal center (GC) B cells, and plasma cells. Moreover, DMOSN@RBD induced not only potent humoral immunity but also Th2-biased cellular immunity along with robust Th1-type cellular immune responses, which are pivotal in restricting SARS-CoV-2 infection. Our work provides a simple and environmentally friendly strategy for synthesizing nanovaccines with significant immunostimulatory potential, offering novel insights for the future development of durable and effective antiviral broad-spectrum nanovaccines.

**IMPORTANCE:**

The persistent evolution of severe acute respiratory syndrome coronavirus-2 (SARS-CoV-2) underscores the critical need to continually assess vaccine immunogenicity and protective efficacy against emerging variants in preclinical animal models. Our study demonstrates that biomimetic nanoparticle vaccines elicit more durable antibody responses and enhanced T cell responses compared to conventional aluminum hydroxide-adjuvanted formulations. Notably, RBD antigen-decorated dendritic mesoporous organosilica nanoparticles (DMOSN@RBD) exhibit broad-spectrum neutralization potential against multiple SARS-CoV-2 variants of concern (VOCs). These findings establish engineered mesoporous silica nanoparticles as a potent immunostimulatory platform capable of simultaneously enhancing both humoral and cellular immunity in subunit vaccine design, particularly through the induction of robust T cell responses typically challenging to achieve with protein-based vaccines.

## INTRODUCTION

The global coronavirus disease 2019 (COVID-19) pandemic caused by severe acute respiratory syndrome coronavirus-2 (SARS-CoV-2) has exerted profound impacts worldwide, causing substantial economic losses and endangering public health (1). The results of hemolysis assays are also evidence of the biosafety of RBD antigen-decorated dendritic mesoporous organosilicon nanoparticles (DMOSN@RBD). The breakthrough infections are primarily driven by the emergence of SARS-CoV-2 variants of concern (VOCs), which have the ability to evade the immune response and compromise vaccine efficacy. Of particular concern is the Omicron variant, which exhibits increased transmissibility and significant evasion of neutralizing antibodies, substantially complicating pandemic containment strategies (2, 3). The emergence of these variants has underscored the importance of reassessing vaccine development strategies to address declining antibody levels and the impact of viral mutations.

Rational adjuvant design integrating antigen presentation with immunomodulatory components represents a critical pathway for next-generation vaccine development (4). Nanoparticle-vaccine platforms, such as lumazine synthase, ferritin, or I53-50 protein assemblies to display SARS-CoV-2 spike or RBD antigens, have shown promise in eliciting potent neutralizing antibody responses (5–8). However, many existing nanovaccines still require co-administration with potent adjuvants to achieve optimal immunogenicity (9–12). Synthetic nanoparticles offer unique advantages through antigen encapsulation or adsorption that enhance antigen stability, cellular internalization, and immunostimulatory potential, positioning them as promising nanoadjuvant candidates (13). DMOSN has garnered significant attention due to its ultrahigh specific surface area, easy surface modification, tunable particle dimensions, excellent biocompatibility, and intrinsic adjuvant properties (14). The strategic incorporation of organic functional groups into inorganic silica (–Si–O–Si–) frameworks enables precise engineering of organosilica nanoparticles for enhanced antigen-presenting cell (APC) activation and effectively improved immune responses (15, 16). However, the application of DMOSN-based systems as nanoadjuvants for SARS-CoV-2 vaccines remains largely unexplored.

In this study, we loaded the SARS-CoV-2 RBD protein into DMOSN to generate a novel biomimetic nanovaccine with self-adjuvant effects. DMOSN@RBD demonstrated the ability to elicit robust cross-neutralizing antibodies in mice and golden hamsters, conferring effective protection against SARS-CoV-2-induced clinical manifestations. Furthermore, the DMOSN-delivered RBD vaccine promoted early dendritic cell (DC) recruitment or activation and induced robust germinal center responses. Additionally, DMOSN@RBD stimulated T-cell-mediated cellular immunity characterized by enhanced cytokine production, while simultaneously maintaining potent humoral immune responses. Overall, this study presents a promising antigen delivery platform for the prevention of epidemics and promotes the application of synthetic biology technology in vaccine production.

## RESULTS

### Construction and characterization of DMOSN@RBD

**Fig 1:**
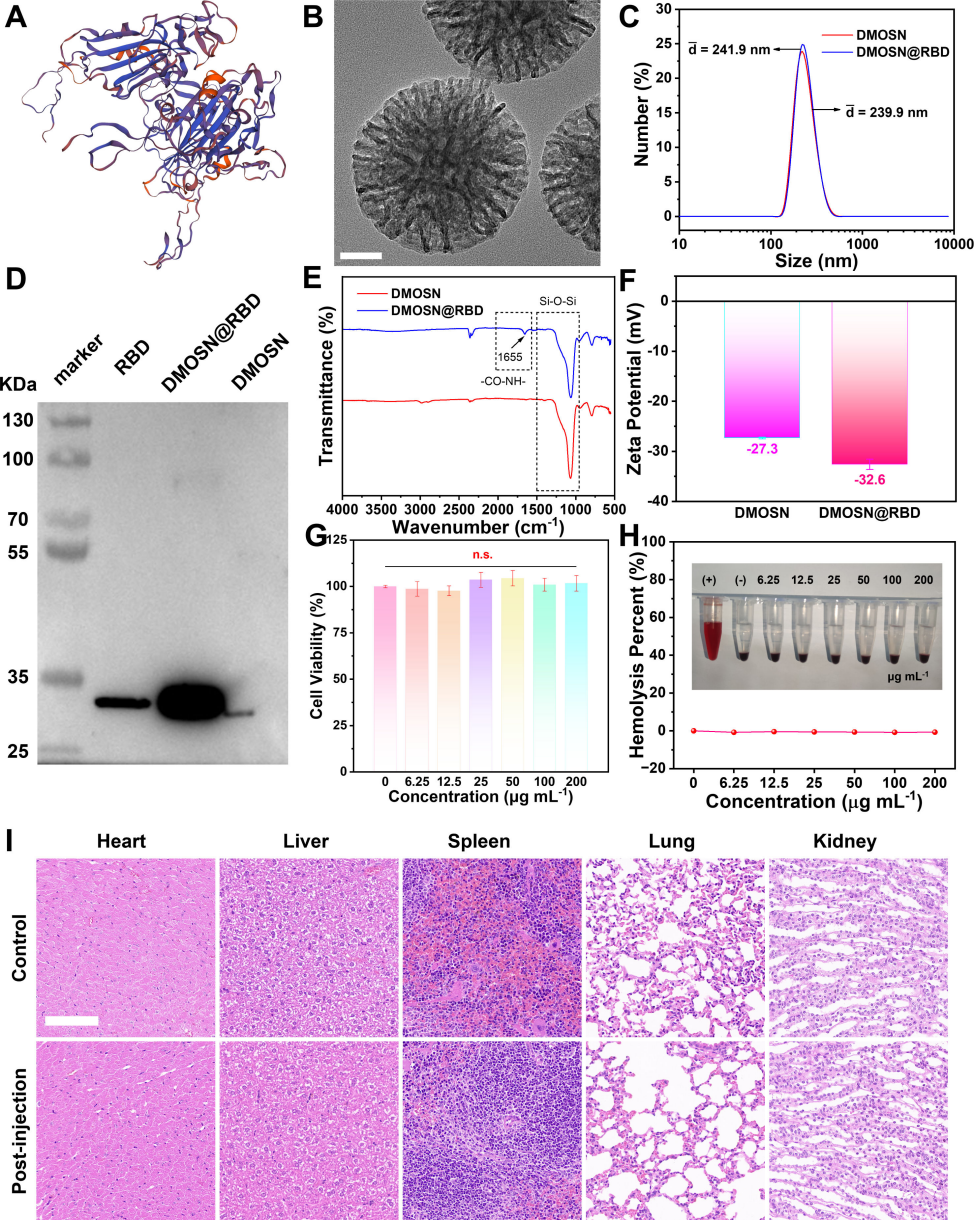
Construction, characterization, and biocompatibility of DMOSN@RBD. (A) Structure of the RBD protein. (B) TEM image of DMOSN (scale bar: 50 nm). (C) Dynamic light scattering (DLS) analysis of DMOSN and DMOSN@RBD. (D) Western blot of RBD, DMOSN@RBD, and DMOSN. FI-IR spectra (E), and zeta potential analysis (F) of DMOSN and DMOSN@RBD. (G) *In vitro* cytotoxicity of different concentrations of DMOSN@RBD to L929 cells after incubation for 24 h (*n* = 4, n.s. based on *t*-test). (H) Hemolysis percentage of red blood cells after incubation with various concentrations of DMOSN@RBD. Inset: digital photos showing the hemolysis effects after 6 h of incubation. Water was used as the positive control (+), and phosphate buffered saline (PBS) was used as the negative control (−). (I) Histological changes in the heart, liver, spleen, lung, and kidney of the mice 28 days after intravenous injection of DMOSN@RBD (scale bars: 100 µm).

The structure of the SARS-CoV-2 RBD protein is shown in Fig. 1A. Subsequently, we loaded RBD proteins into DMOSN pre-prepared by a facile anion-assisted method. Transmission electron microscopy (TEM) characterization revealed monodisperse DMOSN particles with a uniform hydrodynamic diameter of 239.5 nm (Fig. 1B; Fig. S1A and B). Energy-dispersive X-ray spectroscopy suggested that the elements Si (1.75 eV) and O (0.52 eV) were present in the prepared carriers (Fig. S1C). In addition, the X-ray photoelectron spectra (XPS) and high-resolution XPS of the corresponding elements are shown in Fig. S1D, with the peaks attributed to Si 1 s (103.4 eV) and O 1 s (533.0 eV). Furthermore, two nanoparticles were randomly selected for elemental mapping imaging analysis, as displayed in Fig. S1E. Si and O are evenly distributed throughout the particles. Figure S1F showed the nitrogen adsorption-desorption isotherms of DMOSN. The Brunauer-Emmett-Teller surface area was measured to be as high as ~422 m^2^/g. The pore size is estimated to be ~15.3 nm, confirming the mesoporous architecture suitable for vaccine delivery applications.

Therefore, DMOSN and RBD were combined to construct a silicon adjuvant-assisted vaccine carrier composite, DMOSN@RBD. The dynamic light scattering (DLS) analysis of DMOSN and DMOSN@RBD revealed that the hydrated particle sizes were 239.9 and 241.9 nm, respectively, indicating that the RBD did not obviously affect the particle size of DMOSN (Fig. 1C). Western blot analysis showed that the size of RBD monomer was consistent with the size of RBD in the nanoparticles (Fig. 1D). The ultraviolet-visible (UV-vis) absorption spectra confirmed the successful loading of the RBD (Fig. S1G). Fourier transform infrared spectroscopy revealed the characteristic stretching vibrational absorption of “C=O,” which is an amide bond in proteins (Fig. 1E). The change in zeta potential further illustrated the successful construction of the composite structure, DMOSN@RBD (Fig. 1F). In addition, the good sustained-release ability of the nanovaccines for the RBD was also verified (Fig. S1H).

### Biosafety assessment

Cytotoxicity tests and hemolysis assays were performed to assess the biocompatibility of DMOSN@RBD. Mouse fibroblast L929 cells were chosen as the model of normal cells to verify the biosafety of DMOSN@RBD. The cell viability was greater than 95%, even at every concentration (Fig. 1G). The relative intensity of the 576 nm peak is considered the quantitative standard for the percent hemolysis, which is less than 1% at every concentration (Fig. 1H). To verify the long-term toxicity of DMOSN@RBD, one group of healthy mice was injected with DMOSN@RBD, and another group was injected with an equal amount of PBS as a control group. The major organs of the control and injected administration groups were collected after 28 days. As shown in Fig. 1I, there was no significant difference between the control group and the injection group in any organ (heart, liver, spleen, lung, or kidney), indicating the excellent *in vivo* biosafety. The above results demonstrate the low toxicity of DMOSN@RBD.

### DMOSN@RBD vaccination protects mice against lethal challenge by the SARS-CoV-2 mouse-adapted strain

**Fig 2:**
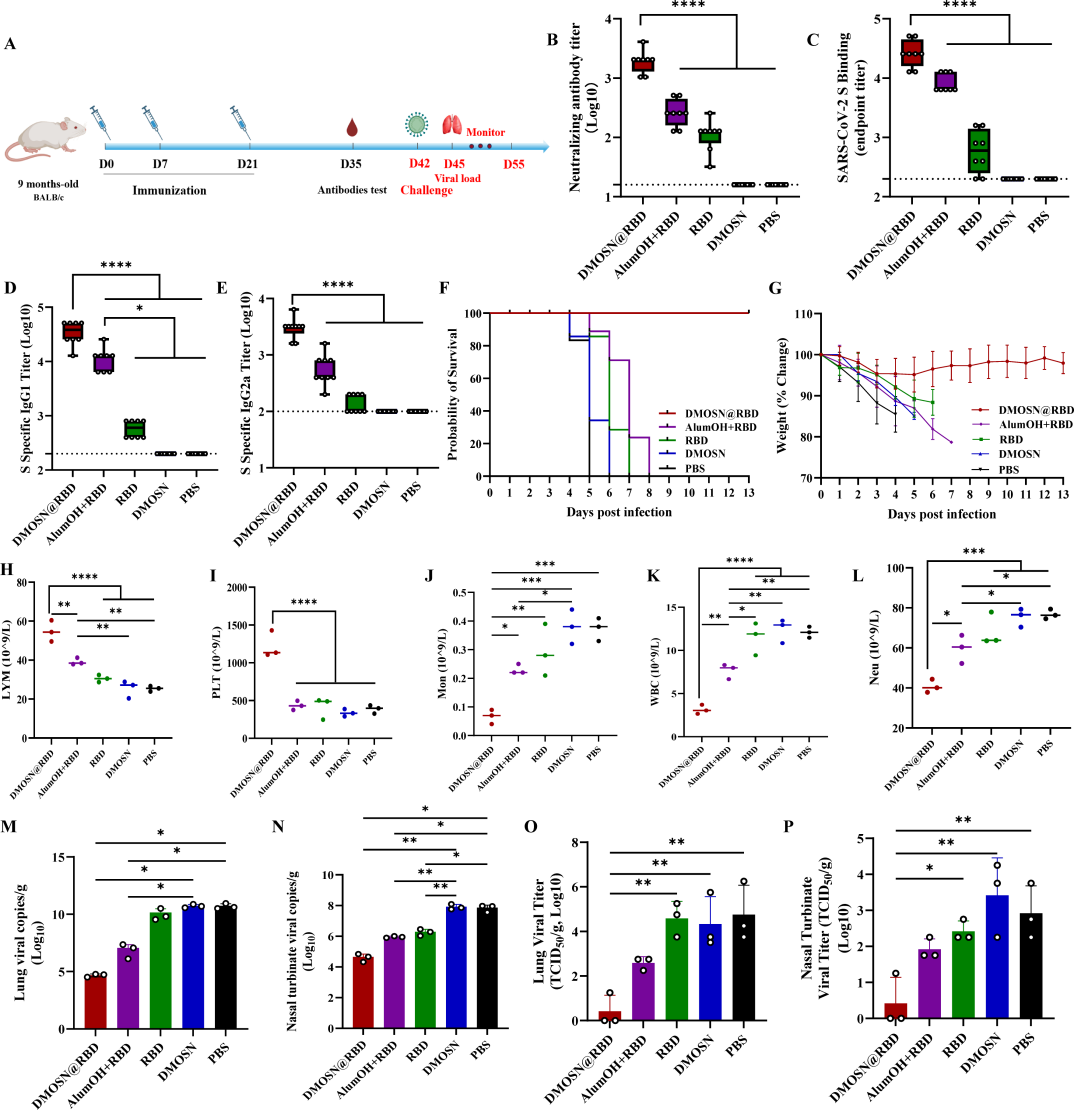
DMOSN@RBD protects mice from SARS-CoV-2 lethal infection. (A) Efficacy schedule. Nine-month-old BALB/c mice (*n* = 10) were immunized via intramuscular injection with DMOSN@RBD, AlumOH+RBD, or RBD on days 0, 7, and 21. At 42 dpi, the mice were intranasally challenged with 10^5^ TCID_50_ of the SARS-CoV-2 mouse-adaptation strain (C57MA14), while unvaccinated and DMOSN-vaccinated mice (*n* = 10) were used as controls. (B) Neutralizing antibody detection at 35 dpv. (C–E) Anti-S-specific IgG (**C**), IgG1 (**D**), and IgG2a (**E**) detection at 35 dpv. (F) The challenged mice were monitored for changes in mortality for 13 days. (G) The challenged mice were monitored for changes in body weights. (H–L) The hematological values of 9-month-old BALB/c mice were analyzed, including LYM% (**H**), PLT count (**I**), Mon% (**J**), WBC count (**K**), and Neu% (**L**) at 3 dpi. (M–P) Lung tissue and nasal turbinates were collected and analyzed for viral RNA load by qRT-PCR and TCID_50_. Groups were compared by one-way analysis of variance (ANOVA) with Tukey’s post-test; ns, not significant, *P < 0.05, **P < 0.01, ***P < 0.001, ****P < 0.0001.

Aging is a high-risk factor for severe COVID-19 (17). The protective effects of DMOSN@RBD vaccination were assessed in 9-month-old female BALB/c mice with a lethal mouse-adapted SARS-CoV-2 strain C57MA14 (18). Nine-month-old female BALB/c mice were divided into five groups receiving intramuscular administration of DMOSN@RBD, aluminum(III) hydroxide (AlumOH)+RBD, the RBD monomer, DMOSN, or PBS, following a prime-boost regimen on days 0, 7, and 21 (Fig. 2A). Neutralizing antibody assay results showed that complete neutralizing antibody titers elicited by DMOSN@RBD were significantly higher than those in the AlumOH+RBD or monomeric RBD immunization groups (Fig. 2B). Measurement of IgG titers and IgG subtypes against the SARS-CoV-2 S protein in serum samples using enzyme-linked immunosorbent assay (ELISA) revealed significant increases following DMOSN@RBD and AlumOH+RBD immunization (Fig. 2C through E). Notably, while antibody subtypes elicited by DMOSN@RBD were dominated by IgG1, significantly higher IgG2a levels were observed compared with the AlumOH+RBD or monomeric RBD immunization groups, indicating a stronger Th1 and Th2 response induced by DMOSN@RBD (Fig. 2D and E). In contrast, immunization with the DMOSN or PBS showed negligible immunogenicity across all parameters (Fig. 2B through E). At 42 days post-vaccination (dpv), the SARS-CoV-2 mouse-adaptation strain C57MA14 was intranasally inoculated with 10^5^ TCID_50_, body weight changes and survival rates were monitored for the subsequent 13 days, and blood biochemical analyses, as well as viral load determinations in the lungs and nasal turbinates, were performed at 3 days post-infection (dpi). The results demonstrated that mice inoculated with DMOSN@RBD exhibited a 100% survival rate and maintained stable body weight throughout the observation period (Fig. 2F and G). In contrast, all animals in the other immunization groups experienced rapid weight loss and succumbed to infection within 8 dpi (Fig. 2F and G). Next, we examined changes in blood cell counts, growth kinetics, and viral loads in the five groups of mice at 3 dpi. DMOSN, PBS, or monomer RBD-immunized mice exhibited increased white blood cells (WBCs), decreased platelets (PLTs), a significant decrease in the percentage of lymphocytes (LYM%), a significant increase in the percentage of neutrophils (Neu%) and monocytes (Mon%) (Fig. 2H through L), which is consistent with the characteristics of SARS-CoV-2 infections (19). Furthermore, changes in LYM, PLT, Mon, WBC, and Neu in the DMOSN@RBD-immunized group were significantly superior to those in the AlumOH+RBD group (Fig. 2H through L). At 3 dpi, three mice from each group were euthanized, and lung and nasal turbinate tissue samples were collected for viral copy analysis and virus titer determination. Animals vaccinated with three doses of DMOSN@RBD demonstrated significant inhibition of SARS-CoV-2 replication compared to the high-level viral load observed in the RBD monomer, DMOSN, or PBS immunization group (Fig. 2M through P). Viral loads in tissues of DMOSN@RBD-immunized mice were slightly lower than those in the AlumOH+RBD group, with notably reduced titers observed in lung and nasal turbinate samples. Specifically, two animals in the DMOSN@RBD group showed viral titers below the detection limit (Fig. 2O and P). These findings demonstrate the high efficacy of DMOSN@RBD in controlling SARS-CoV-2 morbidity, lethality, and virus replication in a mouse model of infection.

### DMOSN@RBD induced cross-neutralizing antibodies and protected against challenges in golden hamsters

**Fig 3:**
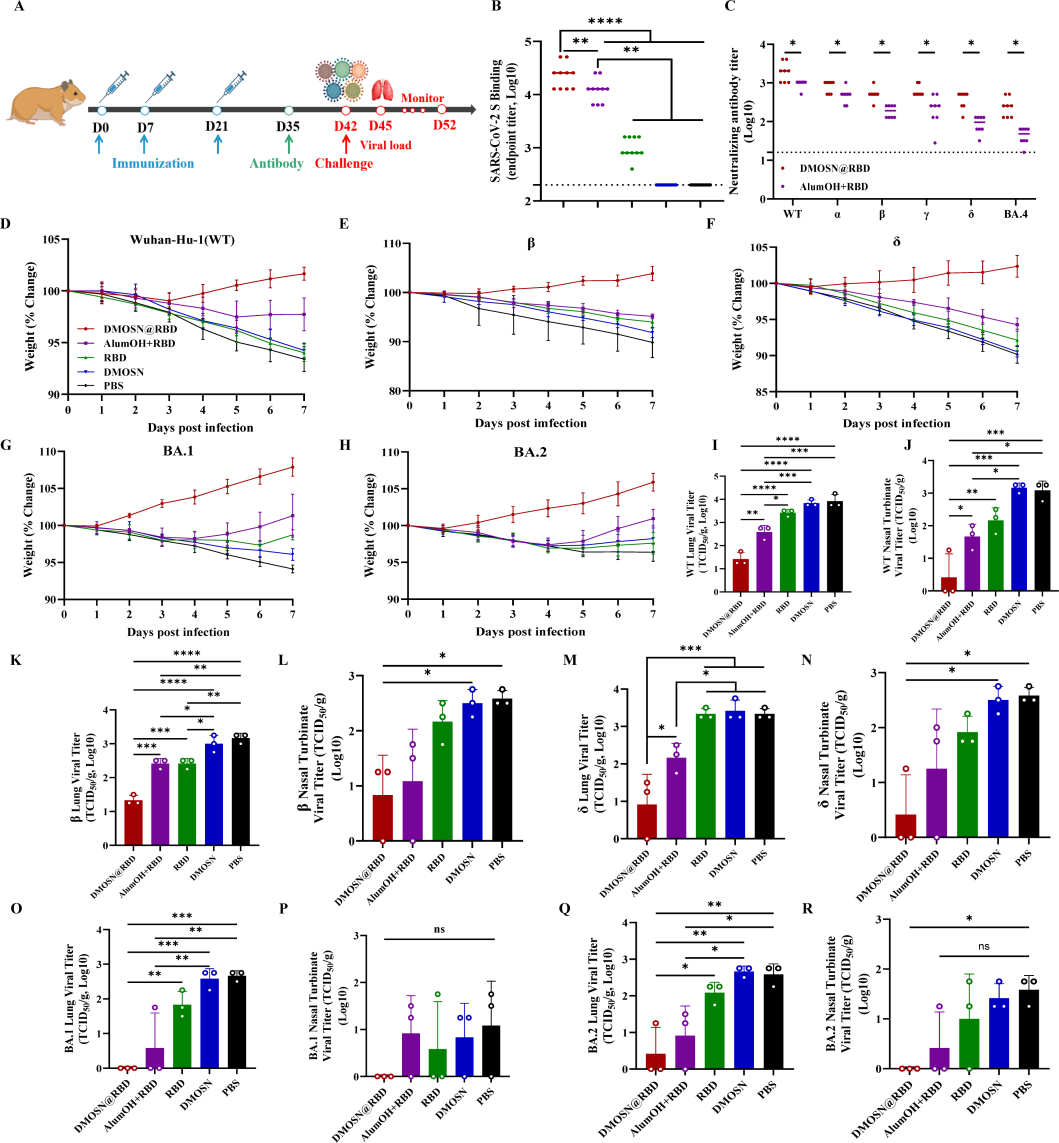
DMOSN@RBD induces a broad-spectrum protective response against SARS-CoV-2 in golden hamsters. (A) Experimental outline. Golden hamsters were immunized with DMOSN@RBD, AlumOH+RBD, the RBD monomer DMOSN, or PBS on days 0, 7, and 21 (*n* = 10) or remained nonimmunized (*n* = 10). All animals were challenged at 42 dpv, and blood samples were obtained on the specified days. The animals were euthanized at 7 dpi. (B) SARS-CoV-2 S-specific IgG titers were detected by. (C) Neutralizing antibodies were detected by neutralization assays. (D–H) Body weight relative to 0 dpi, the symbols indicate means, and error bars represent SD, and the solid line represents 100%. (I–R) Infectious virus titers at 3 dpi in the lungs and nasal turbinate bone after challenge with SARS-CoV-2 Wuhan-Hu-1 (WT) or its variants. Groups were compared by one-way ANOVA with Tukey’s post-test; ns, not significant, *P < 0.05, **P < 0.01, ***P < 0.001, ****P < 0.0001.

Gold hamsters are susceptible to SARS-CoV-2 infection and develop the disease (20). To evaluate the broad protective efficacy of DMOSN@RBD, we utilized this animal model. Following an immune strategy similar to that used for BALB/c mice, we measured neutralizing antibodies and spike protein-binding antibodies at 35 dpv (Fig. 3A). Consistent with the results in mice, compared to the AlumOH+RBD immunization group, DMOSN@RBD elicited higher levels of cross-neutralizing antibodies and S protein-binding antibodies in golden hamsters (Fig. 3B and C). At 42 dpv, golden hamsters were infected with SARS-CoV-2 Wuhan-Hu-1 (wild type, WT) and its variants (Beta, Delta, BA.1, and BA.2) (10^7^ TCID_50_) to evaluate the broad protective effect of DMOSN@RBD. Following the challenge, AlumOh+RBD-, RBD monomer-, DMOSN-, or PBS-immunized animals developed clinical disease with progressive body weight loss. In contrast, DMOSN@RBD-immunized animals were shielded from clinical disease and body weight loss (Fig. 3D through H).

Furthermore, DMOSN@RBD immunization resulted in a significant reduction in the viral load in both the upper and lower respiratory tract (Fig. 3I through R; Fig. S2). A comparison of the titers of infectious viruses in the nasal turbinates and lungs revealed that immunization of golden hamsters with DMOSN@RBD had remarkable protective effects (Fig. 3I through R). Compared with golden hamsters vaccinated with AlumOH+RBD or the RBD monomer, those vaccinated with DMOSN@RBD demonstrated more than 10-fold reduction in viral load in their nasal turbinates and lungs (Fig. 3I through R). Notably, the DMOSN@RBD immune group showed no detection of live virus in the nasal turbinates after challenge with Omicron BA.1 or BA.2 (Fig. 3O, P and R). These findings are consistent with the broad neutralization data, demonstrating that immunogens based on the SARS-CoV-2 WT strain have similarly strong neutralizing effects against the beta, delta, and omicron BA.1 and BA.2 variants. This indicates that DMOSN, in conjunction with an adjuvant for RBD proteins, can induce a strong, broad immune response against SARS-CoV-2 WT and its variants.

### DMOSN@RBD enhances lymph node targeting and elicits potent immune activation

**Fig 4:**
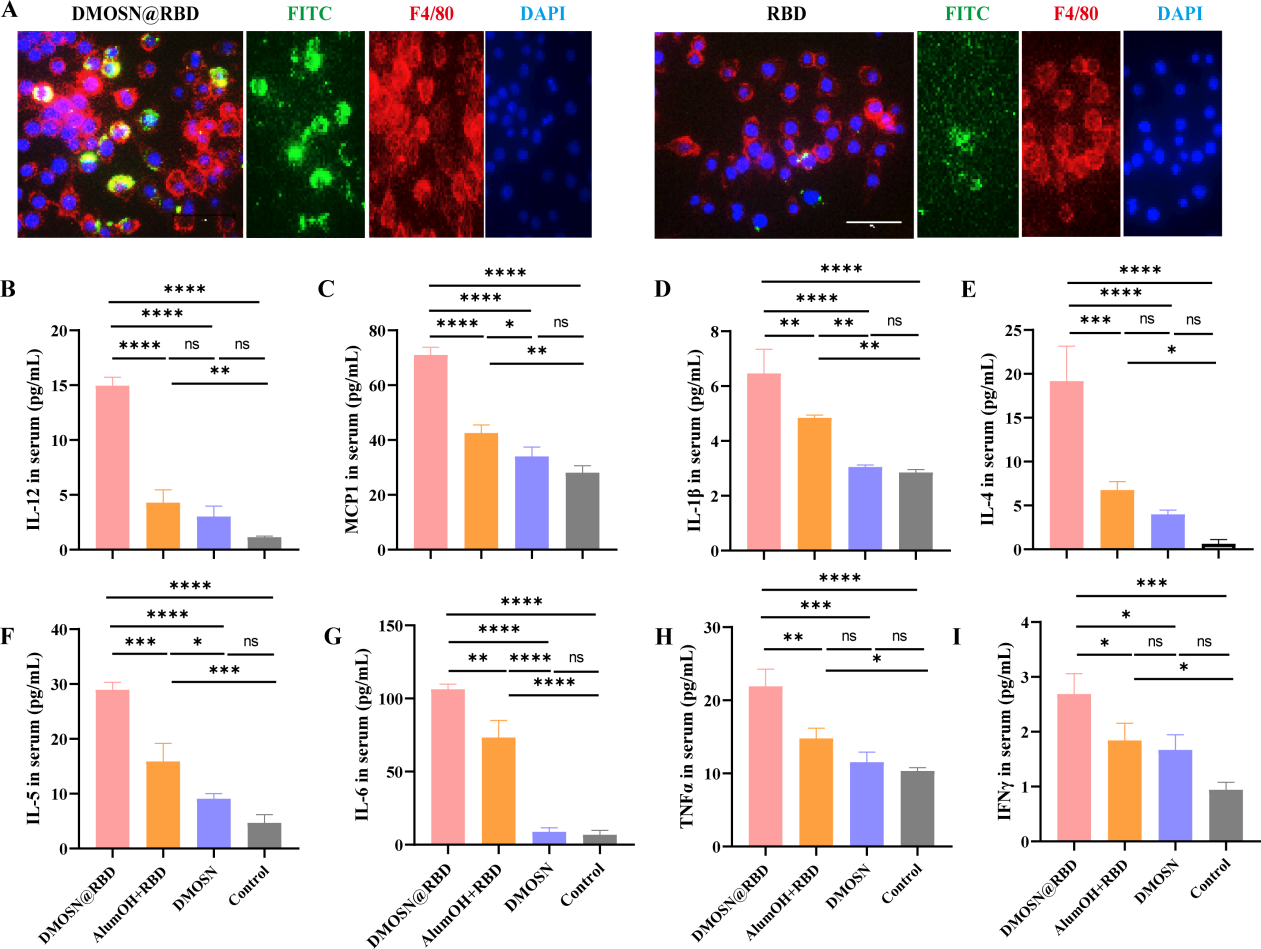
Cellular uptake and antigen presentation of DMOSN@RBD. (A) Immunofluorescence staining showing RAW264.7 cell uptake of DMOSN@RBD and RBD monomers. Green represents FITC-labeled RBD display, red represents F4/80-labeled RAW264.7 cells, and DAPI is blue. The scale bar is 50 µm. (B–I) The release spectrum of cytokines, including IL-12p70 (**B**), MCP1 (**C**), IL-1β (**D**), IL-4 (**E**), IL-5 (**F**), IL-6 (**G**), TNF-α (**H**), and IFN-γ (**I**). Groups were compared by one-way ANOVA with Tukey’s post-test; ns, not significant, *P < 0.05, **P < 0.01, ***P < 0.001, ****P < 0.0001.

Efficient and rapid antigen uptake and activation of antigen-presenting cells are critical for vaccine-induced adaptive immune responses. To clarify whether DMOSN accelerates antigen uptake by APCs, we labeled the RBD protein with fluorescein isothiocyanate (FITC) and assessed the internalization efficiency of DMOSN@RBD in DC2.4 and RAW264.7 cells. The results showed that encapsulating the RBD protein within DMOSN significantly enhanced the internalization of RBD protein in both DC2.4 and RAW264.7 cells (Fig. S3A and B). Immunofluorescence staining also showed that RBD proteins were internalized by macrophages more rapidly than the RBD monomers (Fig. 4A).

Next, we examined the activation of DMOSN@RBD on DCs in lymph nodes *in vivo*. As a benchmark, we vaccinated animals with RBD protein emulsified in aluminum(III) hydroxide (alumOH), which is the most widely used adjuvant (21). DC activation in dLNs was detected at 24 h post-injection, and flow cytometry results revealed that DMOSN@RBD significantly increased the expression of the costimulatory molecules CD86 and CD40 (Fig. S3C and D), as well as that of MHC II, which is essential for T-cell recognition (Fig. S3E), on the surface of DCs. RBD+alumOH only induced elevated MHC II expression, with no significant changes in CD86 and CD40, and its activation efficiency was considerably lower than that in the DMOSN@RBD-immunized group (Fig. S3C through E). These findings clearly demonstrate that utilizing the DMOSN platform for antigen packaging successfully improves antigen presentation efficiency in mice. Considering the immunostimulatory effects observed following DMOSN@RBD injection, we conducted a multicytokine analysis. The results revealed an enrichment of IL-12p70, MCP1, and IL-1β in DMOSN@RBD-immunized groups (Fig. 4B through D). Additionally, a significant increase in IL-4, IL-5, and IL-6 (Fig. 4E through G), accompanied by a slight increase in TNF-α and IFN-γ (Fig. 4H and I), indicated activation of the adaptive immune response. In contrast, the AlumOH+RBD group showed minimal elevation of T cell immune-related molecules (IFN-γ, TNF-α, and IL-2), and the levels of IL-12p70, MCP1, IL-4, IL-5, and IL-6 were significantly lower than those in the DMOSN@RBD group (Fig. 4B through I; Fig. S3F and G). These results emphasize that DMOSN@RBD activates the immune response at an early stage.

### DMOSN@RBD elicits potent humoral immune responses

**Fig 5:**
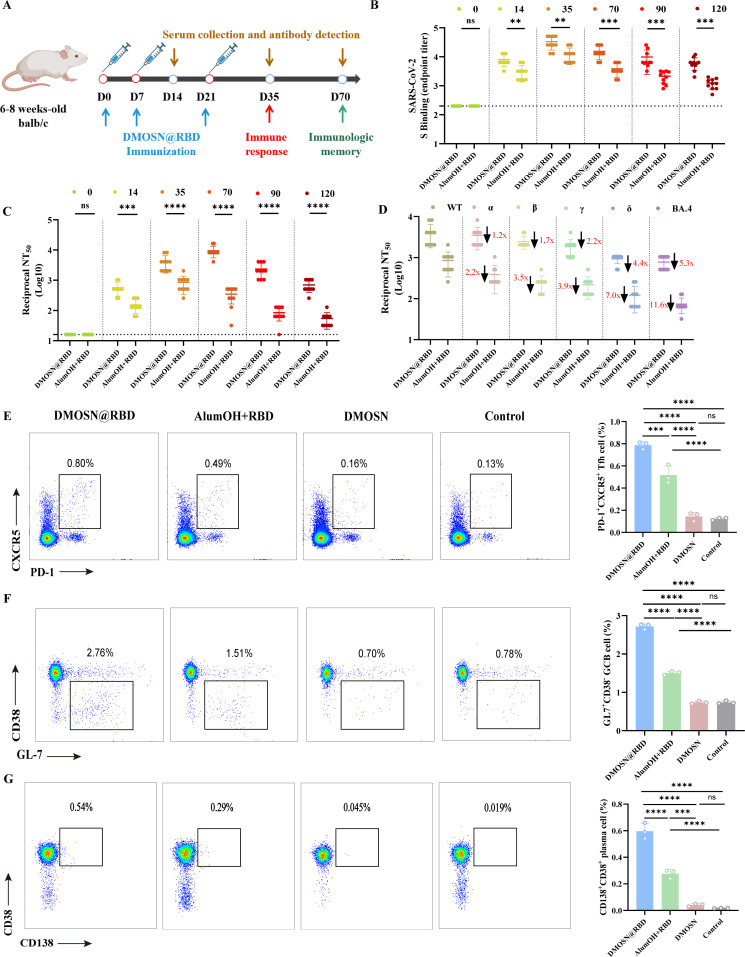
DMOSN@RBD activation of the germinal center response. (A) Immunization scheme. Six- to eight-week-old BALB/c mice were inoculated three times by intramuscular injection. Samples were collected at the indicated time points. (B, C) IgG titers (**B**) and neutralizing antibody titers (**C**) of immunized BALB/c mice. Sera were collected at 0, 14, 35, 70, 90, and 120 dpv for detection. (D) Complete neutralization antibody values of the WT, B1.1.7, B.1.351, P.1, B.1.617.2, and BA.4 viruses at 35 dpv. Mice in the immune group were vaccinated with DMOSN@RBD, alumOH+RBD, or DMOSN. Control animals were inoculated with PBS. (E–G) TFH (CD4^+^CXCR3^+^PD-1^+^) cell (**E**), GCB (CD19^+^GL7^+^CD38^−^) cell (**F**), and plasma (CD19^+^CD38^+^CD138^+^) cell (**G**) responses after DMOSN@RBD, alumOH+RBD, DMOSN, or PBS immunization. A representative flow cytometry scatter plot (left) and a bar chart representing the proportion of cells (right) are shown. The gates for Tfh cells, GC B cells, and plasma cells are shown in Fig. S4, which are based on CD4^+^ T cells and CD19^+^ cells, respectively. The data were combined from two independent experiments. Paired-samples *t*-tests or groups were compared by one-way ANOVA with Tukey’s post-test. The data are presented as the mean ± SD. *P < 0.05, **P < 0.01, ***P < 0.001, ****P < 0.0001; ns, not significant.

To evaluate the role of DMOSN in activating immune responses against SARS-CoV-2, we investigated the humoral and cellular immune responses induced by DMOSN@RBD. Six- to eight-weeks-old BALB/c mice were immunized with an equal amount of RBD protein in the presence of DMOSN or alumOH as adjuvants, while control animals received PBS immunization (Fig. 5A). The DMOSN@RBD group exhibited significantly higher IgG titers than the alumOH adjuvant group (Fig. 5B). Moreover, consistent with the trend observed in anti-S specific IgG detection, the DMOSN@RBD group consistently exhibited higher levels of neutralizing antibodies than the alumOH adjuvant group from 14 to 120 dpv (Fig. 5C). In terms of antibody duration, at 120 dpv, the mice of the DMOSN@RBD group maintained neutralizing antibodies at 2.1 Log, whereas the alumOH adjuvant group exhibited a decrease to less than 20 (Fig. 5C). Furthermore, we evaluated serum neutralizing antibody activity against several pseudotyped viruses from VOCs and a series of Omicron mutants. Our results demonstrated that DMOSN@RBD induced neutralizing antibodies not only against the WT pseudotyped viruses but also against the other five primary VOCs (Fig. 5D). All groups showed a slight reduction in the levels of neutralizing antibodies against B.1.1.7 (Alpha), B.1.351 (Beta), and P.1 (Gamma), whereas the reduction was more significant against B.617.2 (Delta) and BA.4 (Omicron) (Fig. 5D). Notably, the decrease in neutralizing antibodies induced by DMOSN@RBD against each mutant was significantly lower than that observed in the alumOH adjuvant group, especially against Delta and BA.4 (Fig. 5D). Since germinal centers (GCs) in lymph nodes are central to the humoral immune response (22), we further investigated whether DMOSN@RBD could elicit robust GC responses. As expected, at 35 dpv, DMOSN@RBD induced the greatest populations of follicle helper T (Tfh) cells, GCB cells, and plasma cells (Fig. 5E through G; Fig. S4), which was consistent with the detection of high-titer neutralizing antibodies. Given the rapid mutation of SARS-CoV-2 and the problem of rapid decline in neutralizing antibodies against mutant strains with existing vaccines, the significance of the limited decrease in neutralizing antibodies induced by DMOSN@RBD is clearly evident.

### DMOSN as an adjuvant induces strong cellular immune responses

**Fig 6:**
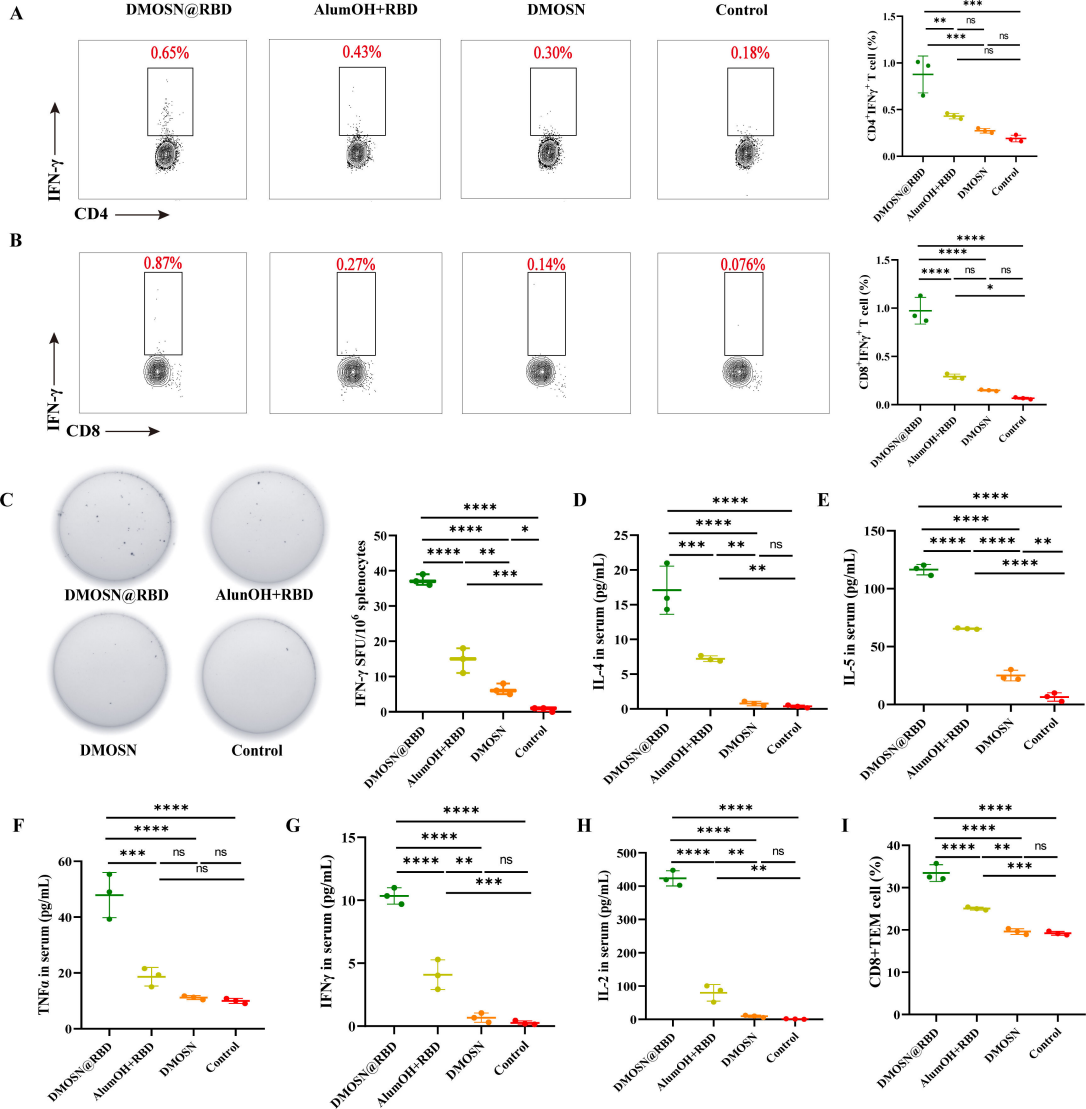
DMOSN@RBD induces antigen-specific T-cell responses. (A, B) Antigen-specific CD4^+^IFN-γ^+^ (**A**) and CD8^+^IFN-γ^+^ (**B**) T-cell responses in the spleen at 35 dpv as measured by flow cytometry. (C) Numbers of IFN-γ-secreting T cells in the splenocyte population were measured using an IFN-γ ELISpot assay. SFU, spot-forming unit. (D–H) The release spectrum of cytokines, including IL-4 (**D**), IL-5 (**E**), TNF-α (**F**), IFN-γ (**G**), and IL-2 (**H**). (I) The percentage of CD8^+^ memory T cells (CD3^+^CD8^+^CD44^+^CD62L^+^) in the spleen at 70 dpv. The data were combined from two independent experiments. Groups were compared by one-way ANOVA with Tukey’s post-test, and the data are presented as the means ± SDs. *P < 0.05, **P < 0.01, ***P < 0.001, ****P < 0.0001; ns, not significant.

Next, we evaluated systemic T-cell activation induced by the vaccines. Splenocytes were stimulated with SARS-CoV-2 S1 and S2 peptides, and flow cytometry analysis was performed after 48 h. The results showed that both the DMOSN@RBD- and alumOH+RBD-immunized groups elicited CD4^+^ IL4^+^ TH2-biased immune responses (Fig. S5A and B), which were consistent with previous studies on subunit vaccines. Interestingly, after the third vaccination with DMOSN@RBD, there was a significant increase in the frequency of antigen-specific CD4^+^ TH1 cells, whereas no increase in IFN-γ^+^ CD4^+^ T cells was detected in the alumOH+RBD-immunized group (Fig. 6A). Moreover, DMOSN@RBD significantly enhanced the secretion of IFN-γ by CD8^+^ T cells (Fig. 6B). The results of the enzyme-linked immunospot (ELISpot) assay were consistent with the flow cytometry results; it demonstrated that DMOSN@RBD induced higher levels of IL-4 and IFN-γ compared to the alumOH adjuvant (Fig. 6C; Fig. S5C). To evaluate the functionality and polarization of antigen-specific T cells, we examined the cytokines secreted by splenocytes in response to S1 and S2 polypeptide stimulation. In DMOSN@RBD-immunized mice, both TH1 (TNF-α, IFN-γ, and IL-2) and TH2 (IL-4 and IL-5)-associated cytokines were significantly elevated; however, TH1-associated cytokines were not increased slightly in alumOH+RBD-immunized mice (Fig. 6D through H). These findings suggest that DMOSN@RBD vaccination induces a more balanced TH1 and TH2 immune response. Additionally, we characterized the long-term memory T cells induced by the DMOSN@RBD vaccine. Our results showed that immunization with DMOSN@RBD vaccine significantly induced the generation of TEM (CD44^+^CD62L^−^) in CD8^+^ T cells (Fig. 6I; Fig. S6A), but there was no significant advantage of DMOSN@RBD in CD4 TEM (Fig. S6B and C). These results highlight that DMOSN, as an adjuvant, effectively delivers the antigen while stimulating a robust humoral immune response and cellular immune response. It compensates for the limitations of subunit vaccines, which may lead to an imbalance between cellular immune responses, thus assisting the body in combating viral infections.

## DISCUSSION

DMOSN have been widely used as delivery vehicles for small-molecule nucleic acid and protein drugs. Recently, they have also gained attention as vaccine delivery systems (14). In this study, we successfully developed a self-adjuvanting SARS-CoV-2 biomimetic nanovaccine employing DMOSN as a delivery system. DMOSN@RBD demonstrated potent broad-spectrum protection against multiple SARS-CoV-2 mutant strains. Furthermore, DMOSN enhanced the internalization of the antigen by macrophages and DCs. Specifically, within 24 h after immunization with DMOSN@RBD, it led to the recruitment and activation of DCs, which in turn resulted in the amplification of antigen-specific humoral and T-cell immune responses. In summary, the preparation process of the DMOSN@RBD biomimetic nanovaccines is straightforward and environmentally friendly. It also exhibits remarkable capabilities in activating both humoral and cellular immunity. The DMOSN-based delivery system holds great potential for delivering various antigens and constructing universal nanoadjuvant vaccines, thus presenting a novel approach for vaccine design.

Subunit vaccines are weakly immunogenic, which necessitates the addition of adjuvants to achieve higher immunogenicity and reduce the required antigen dosage (23). Therefore, the development of an efficient antigen delivery system that can effectively integrate humoral and cellular immunity is crucial for adequate immune protection. Among the various virus-inspired nanocarriers, silica-based nanoparticles have been widely used *in vivo* studies, owing to their unique advantages such as excellent biocompatibility, versatile surface chemistry, and structural stability (24–26). Additionally, silica nanomaterials are biodegradable, capable of slowly dissolving in aqueous solutions to release nontoxic silicic acid, and can be rapidly cleared *in vivo*. This characteristic significantly enhances their biosafety in practical applications (27, 28). In our study, we employed the SARS-CoV-2 RBD protein as an immunogen and formed DMOSN on the surface. This design ensured efficient encapsulation of the antigens, thereby improving their delivery to the host. Notably, compared with traditional alum-adjuvanted vaccines, DMSON@RBD induced higher levels of neutralizing antibodies. This enhanced immune response can be attributed to the fact that the coated silica created a relatively enclosed environment for the RBD protein, reducing its interaction with the aqueous solution and improving the immobilization and reinforcement of the RBD structure (29). Conversely, when the RBD protein was adsorbed by the aluminum adjuvant, there was a significant decrease in the α-helix content, which was compensated by the increase in the β-sheet content. It is hypothesized that the strong ligand exchange between the aluminum adjuvant and the RBD protein may destabilize the protein structure, causing the RBD protein to aggregate on the surface of the aluminum adjuvant and increasing the content of β-sheets. Ultimately, this leads to a reduction in antigen immunogenicity (30).

DCs are the most powerful and only type of APCs capable of activating naïve T cells and initiating the primary immune responses (31, 32). Virus-like mesoporous silica nanoparticles possess a greater capacity for antigen adsorption or internalization compared to silica nanoparticles with a smooth surface or spherical (24, 28). *In vivo* and *in vitro* studies have evidenced the superior ability of DMOSN@RBD to target lymph nodes and recruit and activate DCs in draining lymph nodes. Notably, the activation of DCs represents a pivotal step in triggering T-cell-mediated immune responses induced by DMOSN@RBD. Antigen-specific T cells play an essential role in eliminating infections during the early stages of pathogen transmission and replication (23, 33, 34). On the other hand, although aluminum-based adjuvants are widely used as key components of subunit vaccines (35), alum is not efficient enough in vaccine formulation due to the biased humoral immune response (36). This is particularly evident in scenarios where balanced humoral and T-cell-mediated immune responses are necessary for the prevention of infections and clearance of infected cells (37). These findings indicate that DMOSN has both antigen delivery ability and adjuvant potential, indicating the potential clinical value of DMOSN in vaccine administration.

In conclusion, we demonstrated the potential of DMOSN as a multifunctional nanoadjuvant for recombinant subunit vaccines. Additionally, we have further illustrated that SARS-CoV-2 biomimetic nanovaccines based on DMOSN provide strong, broad-spectrum protection. Specifically, the advantages of DMOSN as a nanoadjuvant for subunit vaccines lie in triggering an effective and longer-lasting antibody response while inducing a potent cellular immune response that confers resistance against SARS-CoV-2 infections. Overall, as a mesoporous nanoparticle, DMOSN holds significant promise for further optimization to enhance vaccination efficacy. As a nanoadjuvant, DMOSN can serve as a versatile platform for vaccine design targeting pandemics and emerging infectious diseases.

## MATERIALS AND METHODS

### Preparation of dendritic mesoporous organosilica nanoparticles

In a typical synthesis, the cationic surfactants CTAB and NaSal were used as structure-directing agents, TEOS and BTEE were used as silica sources, and TEA was a catalyst. First, 450 mg of CTAB and 200 mg of NaSal were added to 30 mL of water and stirred gently at 50°C in an oil bath under magnetic stirring for 2 h. Next, 0.082 g of TEA was added to the above solution and kept stirred for 2 h at 80°C. Then, a mixture of 2.5 mL of TEOS and 2 mL of BTEE was added to the mixture with gentle stirring for 12 h. The products were collected by high-speed centrifugation and washed several times with ethanol to remove the residual reactants. Furthermore, the collected products were refluxed in acetone at 60°C for 6 h five times to remove the template, followed by drying under vacuum at 60°C overnight.

### Characterization of DMOSN

The morphology and composition were studied employing a field-emission SEM (S-4800, Hitachi) instrument equipped with an energy dispersive X-ray spectrometer. TEM images and elemental mapping images were obtained using an FEI Tecnai G2S-Twin instrument with a field-emission gun operating at 200 kV. XPS measurements were obtained using a Thermo Scientific K-Alpha spectrometer. UV-vis absorbance spectra were recorded with a Shimadzu UV-3600 instrument. Infrared spectra were collected with a ThermoFisher Nicolet 6700 infrared spectrophotometer. Determination of zeta potential and hydrated particle size of nanoparticles by Malvern Nanoparticle Size and Potentiometry. The nitrogen adsorption-desorption isotherms were measured by a specific surface area physical adsorption instrument (Micromeritics, ASAP2020M). Cell culture was performed with a Thermo Fisher Incubator.

### RBD loading and release

For the protein loading, the SARS-CoV-2 Wuhan-Hu-1 RBD protein was produced using the Expi293 expression system and dissolved in 0.1 M PBS (pH 7.4) to obtain a protein stock solution with a concentration of 0.2 mg/mL. The RBD solution (5 mL) was added to 10.0 mg of the nanoparticles in 10 mL capped vials. The resulting mixture was shaken at 400 rpm at room temperature for 12 h, followed by centrifugation. The obtained DMOSN@RBD was washed once with PBS and subsequently lyophilized.

For the protein release test, 3.0 mg of nanoparticles after RBD loading were immersed in 1.5 mL of PBS solution and stirred at 200 rpm at 37°C. At predetermined time points, the solution was centrifuged, and the supernatant was removed and replaced with the same amount of fresh PBS solution and used BCA detection kit for protein concentration determination.

### Preparation of FITC-labeled DMOSN@RBD

First, 10 mg of DMOSN@RBD and 0.5 mg of FITC were dissolved in 5 mL of PBS. The mixture was stirred in the dark for 4 h (room temperature, 400 rpm), followed by centrifugation to collect the product, washing twice with PBS, and lyophilization afterward.

### Western blot analysis

The proteins were collected and electrophoresed on 10% SDS-PAGE polyacrylamide gels at 150 V. The proteins were transferred to nitrocellulose membranes for western blotting. The SARS-CoV-2 RBD protein antibody (Sino Biological, China) was used at a 1:3,000 dilution, and goat anti-rabbit IgG (Bioworld, USA) was used at a 1:25,000 ratio. The bands were visualized with SuperSignal West Atto (Thermo Scientific, USA).

### Animal immunization and challenge

Female BALB/c mice aged 6–8 weeks (*n* = 3) were inoculated with DMOSN@RBD via intravenous injection, and various tissues were collected 28 days post-inoculation for pathological analysis.

Female BALB/c mice (6–8 weeks old, *n* = 10) received primary and booster immunizations via intramuscular injection on days 0, 7, and 21. Mice were randomized to receive DMOSN@RBD, AlumOH+RBD, soluble RBD, DMOSN alone, or PBS. Dosing was standardized to 10 µg of RBD per mouse in 100 µL PBS for DMOSN@RBD, AlumOH+RBD, and monomeric RBD groups, with the DMOSN@RBD formulation containing 1 mg of DMOSN carrier. The DMOSN blank control group received an identical DMOSN dosage (1 mg) without RBD conjugation. Biological samples were collected at predefined time points for downstream analyses.

For the challenge of SARS-CoV-2 in 9-month-old BALB/c mice with SARS-CoV-2 at 21 dpv, the mice were inoculated intranasally with a lethal dose (10^5^ TCID_50_) of the mouse-adapted strain C57MA14. All experiments were performed in the BLS-3 laboratory, and clinical signs (body weight, respiratory distress, tremor, and limb paralysis) were observed daily. At 3 dpi, three mice were randomly selected for blood biochemical analysis, followed by the collection of lung, turbinate bone, and serum samples. Animals that survived the infection were euthanized at 13 dpi, and serum was collected.

For immunization and challenge, at 21 days after the booster vaccination, golden hamsters were challenged with 10^5^ TCID_50_ of SARS-CoV-2, including the WT, B.1.351, B.1.617.2, Omicron BA.1, and BA.2 strains. The clinical symptoms and weights of the stimulated animals were continuously recorded for 1 week after infection. At 3 dpi, nasal turbinates and lung tissue were collected for TCID_50_ titration and RNA copy detection.

### Serum neutralization assay

Neutralizing antibody titers against SARS-CoV-2 were quantified via a serum neutralization test with eGFP-expressing vesicular stomatitis virus (VSV) pseudotypes harboring the SARS-CoV-2 S gene. First, heat-inactivated mouse serum was incubated at 56°C for 30 min. Subsequently, the serially diluted serum samples were incubated at 37°C for 1 h with an equal volume of S-pseudotyped VSV particles and inoculated into Vero E6 cells at 37°C with 5% CO_2_ cultivation for 48 h. The neutralizing antibody titer was defined as the reciprocal of the serum dilution required to eliminate all eGFP fluorescence signals. VSV-ΔG-eGFP-SARS-CoV-2 pseudoviruses were preserved in our laboratory. The VSV-△G-eGFP-SARS-CoV-2 pseudoviruses used were genetic SARS-CoV-2 S protein constructs, which included the WT (GenBank: NC_045512.2), Alpha (GISAID: EPI_ISL_601443), Beta (GISAID: EPI_ISL_700428), Gamma (GISAID: EPI_ISL_792680), Delta (GISAID: EPI_ISL_2461258), and omicron BA. 4 (GISAID: EPI_ISL_13360709).

### Enzyme-linked immunosorbent assay

Detection of SARS-CoV-2 anti-S-specific IgG and its subtypes by ELISA. In summary, the SARS-CoV-2 S protein was prepared in our laboratory and then encapsulated in 96-well polystyrene plates overnight at 4°C. After three washes with PBST, 1% bovine serum albumin (BSA, Sigma, Germany) was added to each well for 2 h at 37°C. Next, the mouse or golden hamster serum was added at multiple dilutions and incubated for 1 h at 37°C. After washing three times with PBST, HRP-labeled goat anti-mouse IgG (Bioworld, USA), IgG1, and IgG2a antibodies (Southern Biotech, USA) were added and incubated for 45 min at 37°C. After washing three times with PBST, 3, 3′, 5, 5′-tetramethylbenzidine (TMB, Sigma, Germany) was added for color development, and the reaction was terminated by adding 2 mol/L H_2_SO_4_ at the appropriate time and measuring the OD at 450 nm (Bio-Rad, USA).

### Histopathology and immunohistochemistry

Tissue was fixed in 4% paraformaldehyde, and 3–5 μm paraffin sections were prepared and stained with hematoxylin and eosin for histopathological examination.

### Quantitative reverse transcription-PCR

Tissue or viral RNA was extracted for quantitative reverse transcription-PCR (qRT-PCR) detection as previously described. The collected lung tissue and nasal turbinate were ground with a tissue homogenizer, and the supernatant was collected. Viral RNA was extracted using a Tiangen virus RNA kit (Tiangen, China), and viral RNA was quantified using qRT-PCR targeting the SARS-CoV-2 N gene.

### Viral loads

The supernatants of the nasal turbinate and lung tissue homogenates were serially diluted in DMEM, and then Vero E6 cells were added to 96-well plates. After 72 h of culture at 37°C with 5% CO_2_, the TCID_50_ was detected for cytopathic effects.

### Complete blood cell counts

To determine the complete blood cell counts, samples were analyzed using an auto hematology analyzer (BC-5000vet, Mindray, China) according to the manufacturer’s instructions.

### Mesoscale discovery

For serum collection, blood samples were harvested 7 days after primary immunization. Whole blood was centrifuged at 3,000 × *g* for 30 min at 4°C, and the supernatant serum was collected and stored for subsequent analysis.

For splenocyte supernatant preparation, spleens were isolated 35 days post-immunization, and splenic lymphocytes were separated. Cells were stimulated with the SARS-CoV-2 S1/S2 peptide library for 48 h. Cultured supernatants were then centrifuged at 3,000 × *g* for 20 min at 4°C, and the cell-free supernatants were collected for downstream assays.

The mesoscale discovery assay (Univ#K15048D-X, China) was carried out according to the manufacturer’s instructions. The plates were analyzed on a Sector Imager 2400 system, and cytokine concentrations were calculated based on the standard curve generated in the Discovery Workbench 4.0.12 software with a 4-parameter logistic nonlinear regression analysis.

### Flow cytometry assay

Flow cytometry was performed to detect immune cells in the inguinal lymph nodes. Briefly, at 35 dpv, after the tissue was collected and ground, it was dispersed into individual cells through a 70 mm nylon filter, washed with PBS containing 0.2% BSA, and then treated with a sealing solution containing CD16/32 (Thermo Scientific, USA) for 20 min. A total of 10 (6) cells were stained with fluorescently labeled antibodies, incubated for 30 min, and then washed again. Finally, the stained cells were analyzed using a FACSVerse (Beckman Coulter, USA) instrument.

At 35 dpv, mouse spleen cells were extracted, and the cell concentrations were adjusted to 10^6^/mL with RPMI 1640 (Gibco, USA) culture medium. A lymphocyte proliferation assay was performed with polypeptide (the SARS-CoV-2 S1 [Sino Biological, Cat: PP003-A] and S2 [Sino Biological, Cat: PP003-B] peptide libraries) as the specific stimulator for 12 h, and ConA (500×; Thermo Scientific, USA) as the positive stimulator, as previously described. BFA (1000×; Thermo Scientific, USA) was added to the extracted mouse spleen lymphocytes to block the secretion of intracellular cytokines for 4 h. The cells were then stained with anti-CD3, anti-CD4, and anti-CD8 antibodies, fixed and permeabilized in Fix/Perm buffer, and stained with anti-IL-4 and IFN-γ in Fix/Perm buffer, and flow cytometry was used to detect changes in the cells.

The mouse antibodies used, including CD11c (FITC, N418), CD86 (PE, GL1), CD40 (APC, 1C10), GL-7 (PE, GL-7 [GL7]), PD-1 (PE, J43), CD38 (PerCP-eFluor 710, 90), IFN-γ (APC, 4S.B3), IL-4 (PE, 11B11), CD44 (APC, MEM-263), and CD62L (APC-eFluor 780, MEL-14), were obtained from Thermo Scientific. The antibodies against MHC II (APC/Cyanine7, M5/114.15.2), CD45R (FITC, RA3-6B2), CD4 (FITC, GK1.5), CXCR5 (APC, L138D7), CD8 (PE/Cyanine7, 53-6.7), CD19 (APC/Cyanine7, 1D3/CD19), CD138 (PE, 281-2), and CD3 (APC/Cyanine7, 145-2C11) were obtained from Biolegend.

### *In vitro* cellular uptake

RAW264.7 and DC2.4 cells were seeded at 1 × 10^6^ cells per well in 6-well plates, and uptake experiments were performed until the cells were 80% confluent. The cells were incubated for 4 h in 1 mL of serum-free RPMI 1640 medium containing 5 µg of FITC-RBD or 20 µg of DMOSN loaded with 5 µg of FITC-RBD. Then, the cells were collected, centrifuged, and washed three times with PBS, and antigen uptake was detected using a flow cytometer or indirect immunofluorescence.

### ELISpot

An ELISpot detection kit was used to detect IFN-γ and IL-4 levels (Mabtech, Sweden). According to the manufacturer’s recommended method for testing, a total of 5 × 10^5^ splenic cells were added to each well and treated with 10 µg/mL peptide protein stimulation. The plate was incubated at 37°C with 5% CO_2_ for 36 h. Concanavalin (Thermo Scientific, USA) was used as a positive control. The tablet was scanned on an ImmunoSpot reader. ImmunoSSpot software was used to count specific spots. The number of specific spots/pores must be twice the average value found in each negative control well, and then the background value is subtracted.

## Data Availability

Data used in preparing this article will be released upon request to the corresponding author.

## SUPPLEMENTAL MATERIAL

Supplemental figures jvi.00315-25-s0001.pdf Figures S1 to S6.

ASM does not own the copyrights to Supplemental Material that may be linked to, or accessed through, an article. The authors have granted ASM a non-exclusive, world-wide license to publish the Supplemental Material files. Please contact the corresponding author directly for reuse.
