# Supplementary material for: Biomimetic nanovaccines with self-adjuvant effects induced broad-spectrum neutralizing antibodies against SARS-CoV-2 infection in rodents
Source: J Virol. 2025 Oct 10;99(11):e00315-25. doi: 10.1128/jvi.00315-25 (PMC12645919; doi:10.1128/jvi.00315-25)
Supplement: Supplemental figures — Figures S1 to S6. [file jvi.00315-25-s0001.pdf]

Supporting Information

Biomimetic nanovaccines with self-adjuvant effects inducted broad-spectrum neutralizing antibodies in rodents against SARS-CoV-2 infection

Weiqi Wang[1,3#], Pengye Du[2,3#], Yongkun Zhao[1], Yuan Liang[2,3], Cheng Zhang[1], Hongjie Zhang[2,3], Xianzhu Xia[1], Bo Liu[4], Pengpeng Lei[2,3*], Feihu Yan[1*]*

1 Changchun Veterinary Research Institute, Chinese Academy of Agricultural Sciences, Changchun 130122, China

2 State Key Laboratory of Rare Earth Resource Utilization, Changchun Institute of Applied Chemistry, Chinese Academy of Sciences, Changchun, Jilin, 130022, China

3 University of Science and Technology of China, Hefei 230026, Anhui, China
100071, China

4 Department of Microorganism Engineering, Beijing Institute of Biotechnology, Beijing

# These authors contributed equally.
* Correspondence: Bo Liu, liubo7095173@163.com; Pengpeng Lei. leipp@ciac.ac.cn; Feihu Yan. yanfh1990@163.com.

Supplementary Figure

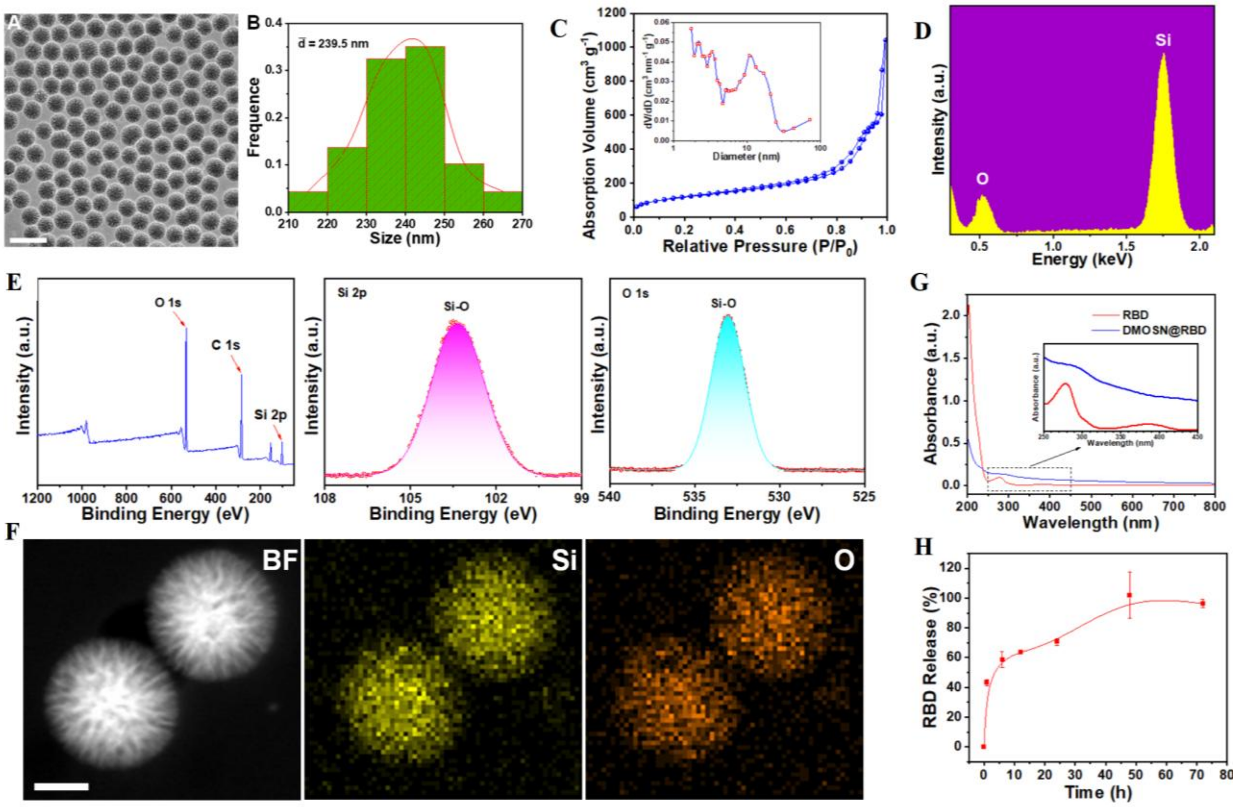

Supplementary Figure 1, related to Figure 1. A, B.TEM image (scare bar: 500 nm) (A) and corresponding size distribution of DMOSN (B). C. Nitrogen adsorption-desorption isotherms, and pore size distribution of DMOSN@RBD (inset). D. EDS spectra of DMOSN. E. XPS spectra: survey spectrum, and high-resolution spectra of Si 2p and O 1s. F. Elemental mapping images of DMOSN (scare bar: 50 nm). G. UV-vis absorbance spectra of RBD and DMOS@RBD. H. RBD release profile as a function of time.

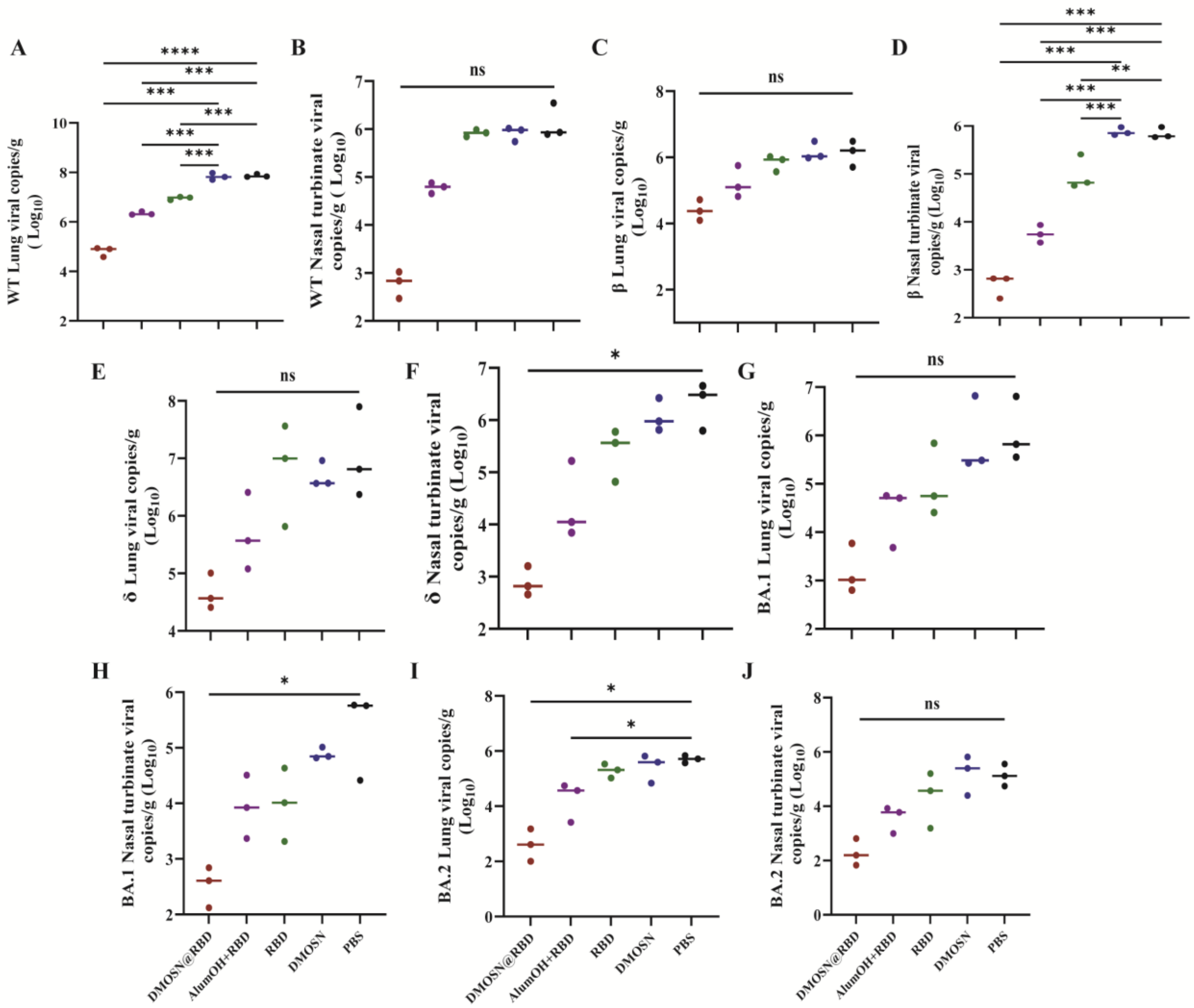

Supplementary Figure 2, related to Figure 3. A-J. Viral copies at 3 dpi in the lungs and nasal turbinate bone after challenge with SARS-CoV-2 Wuhan-Hu-1 (WT) or its variants. Groups were compared by one-way ANOVA with Tukey's post-test, ns, not significant, *$p < 0.05$, **$p < 0.01$, ***$p < 0.001$, ****$p < 0.0001$.

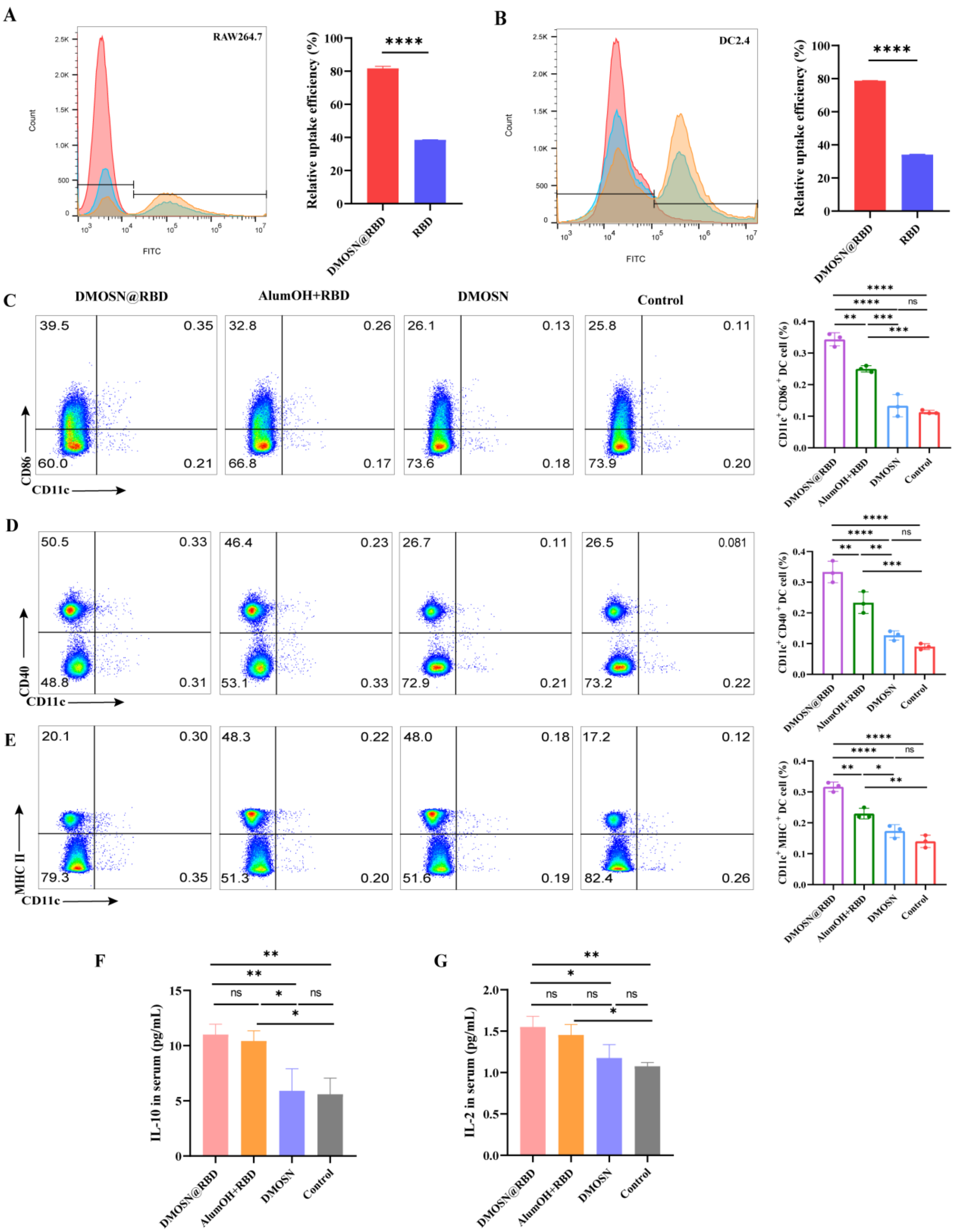

Supplementary Figure 3, related to Figure 4. A-B. Uptake efficiency of DMOSN@RBD by RAW264.7 (A) and DC2.4 (B) cells. C. CD11c^{+} DC cell proportion after DMOSN@RBD, AlumOH+RBD, DMOSN or PBS immunization. A representative flow cytometry scatter plot (left), and a bar chart representing the proportion of cells (right) are shown. C-E. Expression of CD86, CD40 and MHC II on DCs (CD11c+ cell population) in dLNs 24 h post vaccination in mice (n = 3). A representative flow cytometry scatter plot is

shown (left), and the bar chart displays the proportion of cells (right). F-G. The release spectrum of cytokines, including IL-10 (F), and IL-2 (G). Groups were compared by one-way ANOVA with Tukey's post-test, ns, not significant, *$p < 0.05$, **$p < 0.01$, ***$p < 0.001$, ****$p < 0.0001$.

A

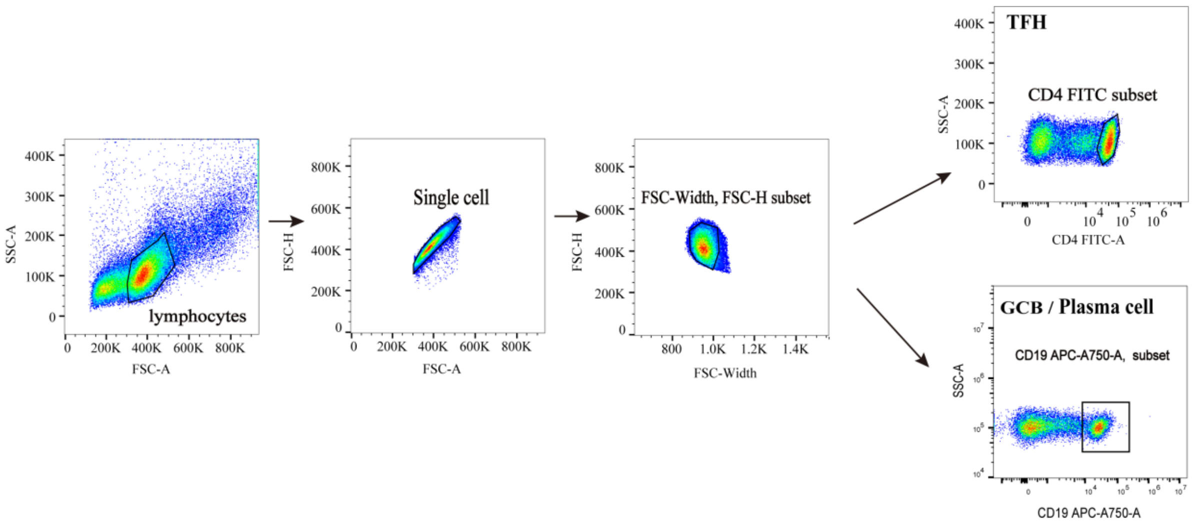

Supplementary Figure 4, related to Figure 5. A. The gating strategies shown are used for $CD4^+$ T cells and $CD19^+$ B cells, respectively, and these gates were applied to analyze the follicular helper T cells (Tfh), germinal center B cells (GC B cells) and plasma cells depicted in Figure 5.

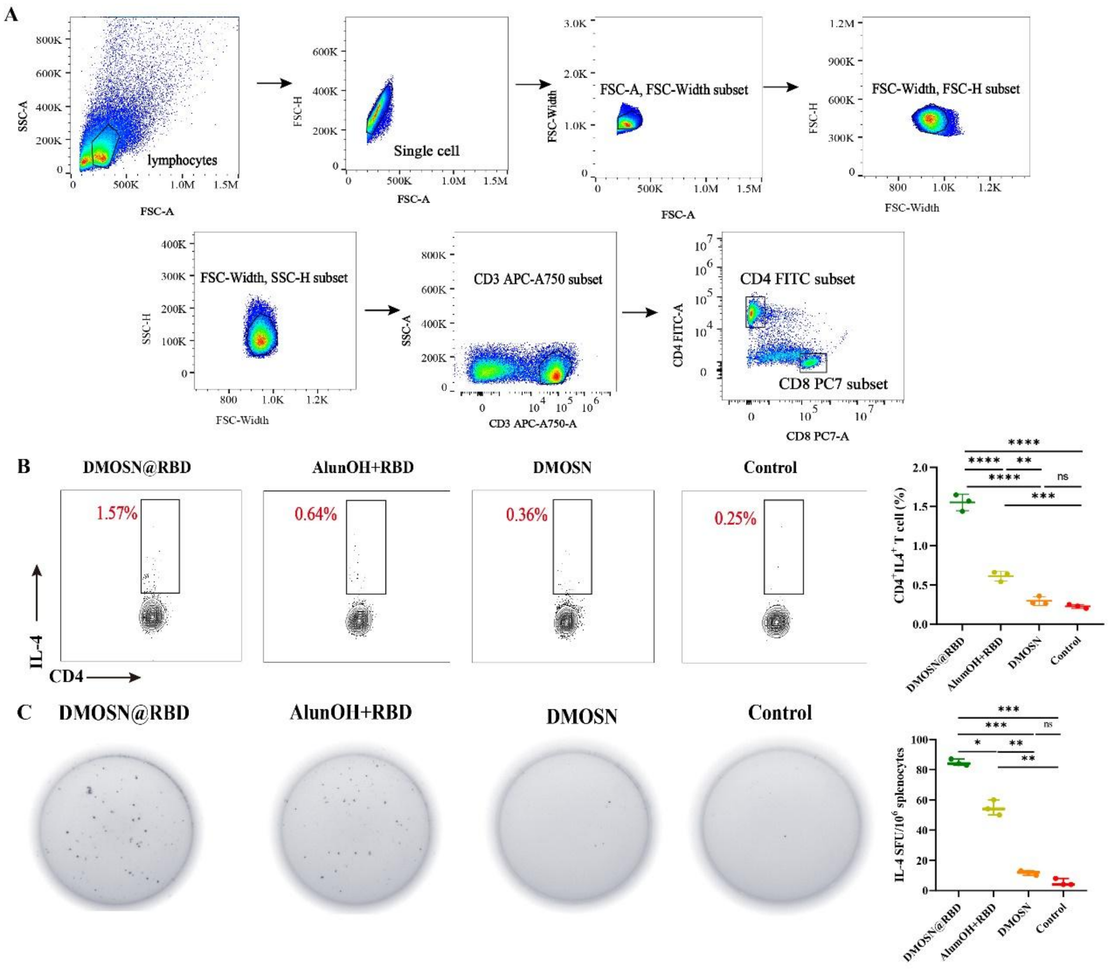

Supplementary Figure 5, related to Figure 6. A. The gating strategy of T cells. B. Antigen-specific CD4^{+}IL-4^{+} T cell responses in the spleen at 35 dpv as measured by flow cytometry. C. Numbers of IL-4 secreting T cells in the splenocytes were measured using IL-4 ELISpot assay. SFU, spot-forming unit. Groups were compared by one-way ANOVA with Tukey's post-test and data are presented as the mean ± SD. *$P < 0.05$, **$P < 0.01$, ***$P < 0.001$, ****$P < 0.0001$, ns, not significant.

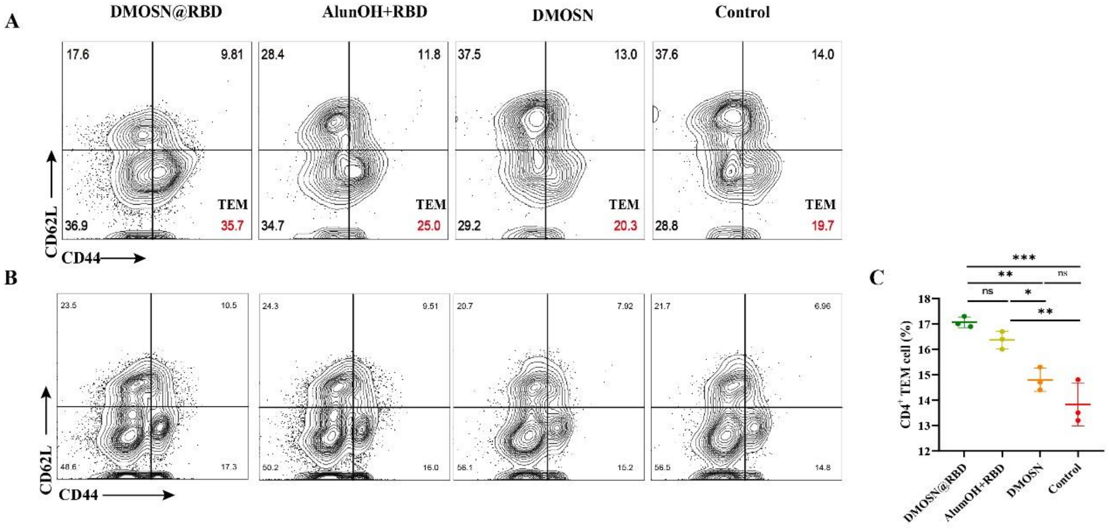

Supplementary Figure 6, related to Figure 6. A. Representative flow cytometry scatter plot of $CD8^+$ memory T cells. B. Representative flow cytometry scatter plot of $CD4^+$ memory T cells. C. The percentage of $CD4^+$ memory T cells. Groups were compared by one-way ANOVA with Tukey's post-test and data are presented as the mean ± SD. *$P < 0.05$, **$P < 0.01$, ***$P < 0.001$, ****$P < 0.0001$, ns, not significant.
